# Executive Functioning as a Pathway Between Social Skills and Psychopathology: The Moderating Role of Autism Diagnosis

**DOI:** 10.1002/aur.70305

**Published:** 2026-06-26

**Authors:** Samina I. Ansari, Lucas A. Martin, Kristi C. Guest, Rajesh K. Kana

**Affiliations:** ^1^ Department of Psychology University of Alabama at Birmingham Birmingham Alabama USA

**Keywords:** autism, behavior regulation, executive functioning, externalizing problems, internalizing problems, metacognitive index, social skills

## Abstract

Internalizing and externalizing problems are highly prevalent among children and adolescents, with higher rates observed particularly among those with neurodevelopmental disorders. Previous studies have identified deficits in social competence and executive functioning (EF) as predictive factors of internalizing and externalizing problems, and some studies have indicated that EF mediates this association in autistic individuals. However, the role of EF and social competence across autistic and typically developing (TD) individuals remains underexplored in relation to internalizing and externalizing behaviors. We address this gap by using two moderated mediation analyses assessing the mediating effects of EF in the association between social skills and internalizing and externalizing problems. Poor metacognitive functioning was found to mediate the relationship between social skill deficits and internalizing problems only among autistic children and adolescents, whereas behavior dysregulation mediated the relationship between poor social skills and externalizing problems among both autistic and TD children and adolescents. These mediating effects of EF, however, were not moderated across the two diagnostic groups. These findings highlight the role of different domains of EF, that is, metacognitive functioning and behavior dysregulation in internalizing and externalizing problems, which can help identify early EF‐related vulnerabilities and inform EF‐based interventions for psychological problems among autistic and TD children and adolescents.

## 1 Introduction

Internalizing and externalizing are two main categories of behavioral problems, with the former describing maladaptive thoughts and behaviors directed towards oneself and the latter manifesting in the context of behavioral interactions with the social environment and directed outwards (Nikstat and Riemann 2020). These are broad categories used for emotional problems, such as withdrawal, anxiety or depression (internalizing problems), and behavioral problems, such as defiant or disruptive behavior and/or attentional problems (externalizing problems) (Achenbach 1966), that are associated with long‐term negative outcomes in adulthood, among autistic individuals as well as the general population (Arslan et al. 2021; Sari et al. 2021). Studies have reported considerably higher prevalence rates of internalizing (24.17%) and externalizing (11.93%) problems among school‐going adolescents (Achenbach et al. 2003; Hunduma et al. 2024). Childhood and adolescence are sensitive years for social development, marked by neuroanatomical and functional changes to adapt to rapid changes in social exposure (Blakemore and Mills 2014; Fuhrmann et al. 2015).

Adaptive and cognitive skills, including social skills and executive functioning (EF), are important in navigating the developmental changes during childhood and adolescence. Social skills are manifestations of social competence, which encompasses a range of skills, including the ability to recognize and integrate social cues, take others' perspectives, and regulate interpersonal expression of responses (Ogden 2015). Social skills are essential for navigating social changes and are associated with favorable developmental and psychological outcomes, including improved academic achievement and psychological well‐being (Bijstra and Jackson 1998; Frogner et al. 2022; González and Fernández 2017; Segrin et al. 2007; Sørlie et al. 2021). On the contrary, a deficit in social communication skills earlier in life is predictive of internalizing and externalizing problems in childhood and adolescence in a community sample (Bornstein et al. 2010). This is particularly evident among individuals with autism spectrum disorder (ASD), where difficulties in social communication and interactions are core clinical symptoms (American Psychiatric Association 2022). Poor social skills are significantly associated with increases in emotional and behavioral problems among autistic children and adolescents (Boettcher et al. 2024; Donoso et al. 2024). Moreover, internalizing and externalizing problems were found to be differentially related to the core autistic symptoms, that is, restricted and repetitive behaviors (RRBs), and social communication difficulties (Tsai et al. 2020). More specifically, externalizing behavior problems, such as inattention and hyperactivity, were moderately associated with RRBs among autistic preschool and school‐aged participants (Tsai et al. 2020). These studies have corroborated a positive relationship between social communication difficulties and internalizing and externalizing problems among both typical and autistic samples. Some researchers propose poor self‐regulation or EF and poor social skills as plausible factors underlying psychological problems among primary school students over time (Wang and Liu 2021).

EF refers to a wide range of higher cognitive abilities (Mezzacappa 2011) and encompasses many skills, including planning, organizing, cognitive flexibility, set shifting, and self‐regulation. Based on the Behavior Rating Inventory of Executive Function (BRIEF), daily life EF can broadly be categorized into two domains: (1) *metacognitive functioning*, which relates to cognitive skills such as planning, organizing, working memory, initiation, monitoring of tasks or one's' behaviors, and (2) *behavior regulation*, which is involved in purposeful regulation of behaviors, and includes skills such as inhibition, shifting and emotion regulation (Gioia et al. 2000). EF has been proposed as a top‐down regulatory process in explaining several developmental psychopathologies, including autism (Nigg 2017; Pennington and Ozonoff 1996). EF is related to social competence in typical individuals (Razza and Blair 2009), and is associated with difficulties in social communication among autistic children (Gilotty et al. 2002). Autistic individuals commonly experience difficulties in EF across their lifespan (Demetriou et al. 2018). The executive dysfunction hypothesis of autism proposes EF difficulties as the key factor behind many autistic symptoms at behavioral and cognitive levels (Hill 2004; Hughes et al. 1994).

Several studies have reported an association between poor EF and internalizing and externalizing problems (Freichel et al. 2024; Wang and Liu 2021; Yang et al. 2022; Zelazo 2020). In a longitudinal study, Fossum et al. (2023) found that autistic adolescents and adults have lower adaptive functioning, and EF is a significant predictor of poor adaptive functioning, particularly among autistic participants compared to typically developing (TD) adolescents and early adults. Studies have also shown poor metacognitive functioning as a mediator between social communication and internalizing problems and difficulties managing daily life tasks among autistic youth (Munsell et al. 2022). Other studies have reported the mediating effect of behavior dysregulation, a component of EF, on the relationship between the severity of core autistic symptoms, including social communication deficits on externalizing problems among autistic participants across different timepoints from childhood through adolescence (Ameis et al. 2022). These studies underscore the importance of examining the mediating role of EF in the relationship between social skills and psychological problems among autistic and TD children and adolescents.

While studies support the role of executive functioning in the association between deficits in social skills and psychological and adaptive difficulties among autistic individuals (Ameis et al. 2022; Munsell et al. 2022), studies exploring such mediating effects in their typical counterparts are limited. Further, it remains unclear whether these mediating effects vary across typical and autistic groups. Early childhood and adolescence are sensitive periods of development with a broader window of plasticity (Selemon 2013). Additionally, early detection of vulnerabilities, and EF‐based targeted interventions have shown to be effective therapeutic and mitigative strategies for symptoms of several psychopathologies (Zelazo 2020).

Therefore, the current study has two goals. The primary goal of this study is to examine the role of EF, that is, metacognitive functioning and behavioral dysregulation in internalizing and externalizing problems among autistic and TD children and adolescents. The study also aims to investigate whether these mediating effects vary across different diagnostic groups, that is, typical and autistic groups. Addressing this gap is important to elucidate how social skills and EF contribute to internalizing and externalizing problems among autistic and typically developing populations, and to determine if there are differential pathways underlying emotional and behavioral problems among autistic and TD participants. This can provide insight into the contributions of social skills and EF in psychological problems, specifically internalizing and externalizing problems, which may enable early identification of vulnerabilities among children and adolescents, and inform the development of EF‐focused interventions for psychological problems among autistic and TD children and adolescents.

## 2 Methods

### 2.1 Participants

Behavioral data from the Autism Brain Imaging Data Exchange‐II (ABIDE‐II) dataset were used in this study (Di Martino, 2016). Participants were included if they were administered the Social Responsiveness Scale (SRS), BRIEF, and Child Behavior Checklist (CBCL) for ages 6–18 years. To ensure comparison across cases, consistent measures of full‐scale intellectual functioning (FIQ) were used. This pool comprised participants across three ABIDE‐II sites: Kennedy Krieger Institute, Georgetown University, New York University (NYU) Langone Medical Center. ABIDE‐II has two discrete samples from the NYU site; both of these were included in the present analysis for a total of four distinct samples.

A final sample of 318 children and adolescents (*M*_age_ = 10.31, SD = 1.31) was retained, where *N*_ASD_ = 122, *N*_TD_ = 196. The participants comprised 70.75% males and 38.36% autistic individuals, with ages of all participants ranging from 6.19 to 14.98 (*M*_age_ = 10.29, SD = 1.57).

### 2.2 Measures

*Social Responsiveness Scale (SRS)*: The SRS (Constantino and Gruber 2005) and SRS‐2 (Constantino and Gruber 2012) measure social ability on a continuum and are normed by sex. For the present study, parent‐report total *t*‐scores for SRS and SRS‐2 were included in the analyses. *Behavior Rating Inventory of Executive Functions (BRIEF)*: The BRIEF (Gioia et al. 2000) was completed by the parent or guardian of the participant. The BRIEF asks questions related to daily living skills and activities that rely on specific EFs. The BRIEF yields an overall Global Executive Composite Score (GEC), which is comprised of the Behavioral Regulation Index (BRI), and the Metacognition Index (MI). The present analysis includes BRI and MI *t*‐scores, where higher scores indicate poor EF. *Child Behavior Checklist (CBCL) for ages 6–18*: The CBCL measures problem behaviors reported by parents (Achenbach and Rescorla 2001). The sex and age‐normed *t*‐scores for internalizing and externalizing problems were used for this study. *Wechsler Intelligence Scale for Children (WISC) and Wechsler Abbreviated Scale of Intelligence (WASI)*: The WISC measures intelligence (full‐scale IQ, FIQ) in children aged 6 years and older, whereas the WASI, a shortened version of WISC, is used for both child and adult samples.

### 2.3 Data Analysis

Prior to the main analyses, preliminary analyses were conducted, including assessment of missingness, outliers, descriptive statistics, and evaluation of statistical assumptions. Assumptions for ordinary least regression, including test of linearity of relationship between predictors and outcome variables, normality of residuals, and homoscedasticity were separately assessed for both models. Bivariate correlation was assessed using Pearson's product‐moment correlation between all observed variables. A follow‐up multicollinearity diagnosis was carried out for both models. Missingness was less than 5%; therefore, listwise deletion was carried out. Although Shapiro–Wilk test revealed that each group was normally distributed for some of the variables and non‐normally distributed for other variables, skewness and kurtosis for the variables within each group were within an acceptable range of ±2. Therefore, independent samples *t*‐tests were conducted to assess group differences in the outcome and predictor variables.

**FIGURE 1:**
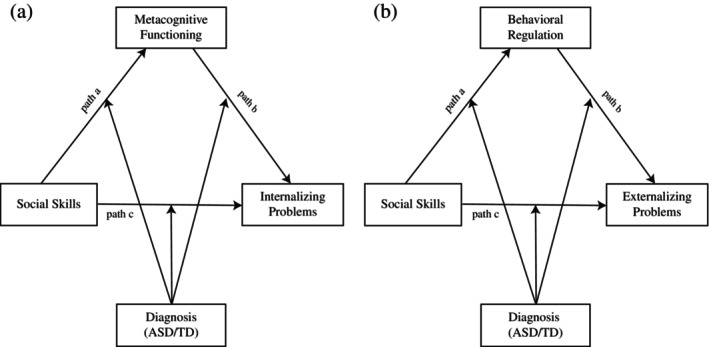
Conceptual Diagram of Moderated Mediation Models. (a) Mediating effects of metacognitive functioning in the association between social skills and internalizing problems, moderated by diagnosis. (b) Mediating effects of behavior regulation in the association between social skills and externalizing problems, moderated by diagnosis. Paths a, b, and c indicate the mediation pathways in the respective models. The arrows pointing towards the pathways indicate the moderation effects of diagnosis groups on each of the pathways. ASD, autism spectrum disorder; TD, typical development.

For main analyses, two separate models using Hayes PROCESS Model 59 (Hayes 2017) were carried out using *R*, one for each outcome variable and corresponding mediator, using a bias‐corrected bootstrap approach (5000 times to estimate 95% confidence intervals). Model 1 examined if metacognition mediates the association between social skills and internalizing problems, and diagnosis was specified as a moderator of direct and indirect effects (see Figure 1). Model 2 examined if behavior regulation mediated the association between social skills and externalizing problems, and if the direct and indirect effects were moderated by diagnosis (see Figure 1). Full‐scale IQ was used as a covariate in both models.

Follow‐up exploratory analyses were conducted using structural equation modeling (SEM) for both Model 1 and Model 2 to examine specific contributions of executive functioning subscales in the association between social skill deficits and psychological difficulties. To this end, each SEM model, that is, SEM Model 1 and SEM Model 2 were specified in two parts, measurement model and structural model.

Measurement models comprised of the latent construct of EF defined by the theoretically derived subscales of metacognitive functioning and behavioral regulation. Thereby, for Model 1, metacognitive functioning was specified as a latent variable indicated by initiation, working memory, planning, organization, and monitoring. In Model 2, behavioral regulation was fitted as a latent variable with inhibition, shifting, and emotional control as its indicators.

In the structural models, social skills were specified as exogenous predictors, and internalizing problems and externalizing problems were specified as endogenous variables for SEM Model 1 and Model 2, respectively. Latent constructs were modeled as variables mediating the association between the endogenous and exogenous variables. Metacognitive functioning was used as a mediator in SEM Model 1 and behavior regulation in SEM Model 2. Direct and indirect effects were estimated simultaneously for each model. Full‐scale IQ was fitted as a moderator for path a and path c′ in both SEM models. However, adding IQ as a moderator yielded poor fits in both SEM models, as indicated by fit indices ([SEM Model 1: CFI = 0.335, TLI = 0.237, RMSEA = 0.255, SRMR = 7.286], [SEM Model 2: CFI = 0.000, TLI = −0.787, RMSEA = 0.355, SRMR = 9.964]). Therefore, IQ was not used as a moderator but was used as a covariate in the final models. The models were estimated using maximum likelihood ratio estimator (MLR), where latent factor loadings and regression coefficients were estimated freely.

## 3 Results

The data were normally distributed, as skewness and kurtosis showed no violations of normality in predictor and outcome variables (See Table 1). The plots of residuals and predicted values indicated that the assumptions of ordinary least regression, including linearity, normality of residuals, and homoscedasticity were not violated for both models. Although the correlation among the predictor variables was high, as shown in Table 2, multicollinearity diagnostics revealed that the assumption of multicollinearity was not violated as indicated by tolerance and variance inflation factor (VIF) for both models (For Model 1: Social skills: Tolerance = 0.356, VIF = 2.810; Metacognitive functioning: Tolerance = 0.346, VIF = 2.891; For Model 2: Social skills: Tolerance = 0.269, VIF = 3.718; Behavior regulation: Tolerance = 0.276, VIF = 3.625; Full scale IQ: Tolerance = 0.944, VIF = 1.059).

**TABLE 1:** Participant characteristics.

	ASD (*N* = 122)		TD (*N* = 196)		Skewness, kurtosis	Independent samples *t*‐test	
	Mean (SD)	Min—Max	Mean (SD)	Min—Max		*t*(316)	*p*
Age (in years)	10.38 (1.78)	6.19–14.98	10.23 (1.43)	6.36–13.65	0.159, −0.511	0.81	0.211
Social skills	77.03 (13.73)	45–116	43.65 (5.71)	34–65	0.905, −0.304	25.52	< 0.001
Behavioral regulation	64.96 (10.35)	39–99	43.61 (7.23)	35–70	0.693, −0.305	19.98	< 0.001
Metacognitive functioning	66.39 (10.20)	37–86	45.18 (8.92)	30–74	0.349, −1.036	19.21	< 0.001
Externalizing problems	56.13 (9.83)	33–77	43.54 (8.39)	32–71	0.180, −0.828	12.18	< 0.001
Internalizing problems	63.72 (9.27)	39–87	46.00 (9.32)	33–71	0.374, −0.617	16.53	< 0.001
FIQ (covariate)	109.31 (17.95)	63–149	116.95 (12.41)	85–149	−0.359, 0.250	−4.48	< 0.001

*Note:* Social skills: Higher scores represent poor social skills; behavioral regulation: Higher scores represent higher behavioral dysregulation; metacognitive functioning: Higher scores represent poorer metacognitive functioning.

Abbreviations: ASD, autism spectrum disorder; FIQ, full‐scale intelligence quotient; TD, typical development.

**TABLE 2:** Bivariate association between predictor and outcome variables.

	1	2	3	4	5	6
1. Social skills	1					
2. Behavior regulation index	0.851***	1				
3. Metacognitive functioning	0.803***	0.816***	1			
4. Internalizing problems	0.729***	0.759***	0.685***	1		
5. Externalizing problems	0.645***	0.765***	0.692***	0.686***	1	
6. Full scale IQ	−0.232***	−0.171**	−0.283***	−0.204***	−0.106	1

*Note:* ***p < 0.001, **p < 0.01 (2‐tailed).

Independent samples *t*‐tests indicated that on average, autistic individuals had significantly higher levels of internalizing problems (*M*_diff_ = 17.72, *t*(316) = 16.53, *p* < 0.001), externalizing problems (*M*_diff_ = 12.59, *t*(316) = 12.18, *p* < 0.001), and poorer social skills (*M*_diff_ = 33.38, *t*(316) = 25.52, *p* < 0.001) compared to TD participants. The average meta‐cognitive functioning (*M*_diff_ = 21.21, *t*(316) = 19.21, *p* < 0.001) and behavior regulation skills (*M*_diff_ = 21.35, *t*(316) = 19.98, *p* < 0.001) were lower among autistic participants compared to TD participants (See Table 1).

**FIGURE 2:**
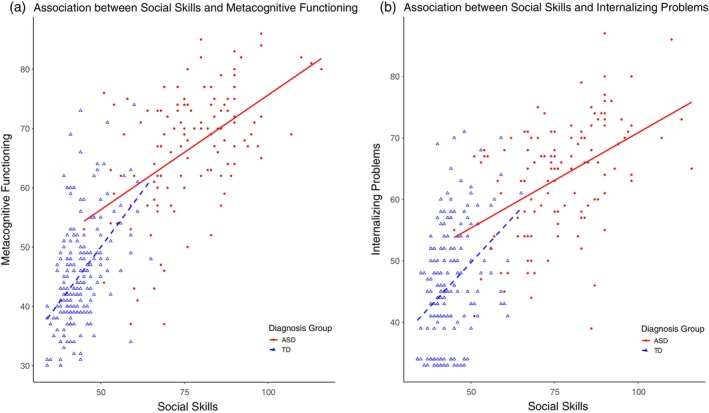
Simple slopes between social skills and metacognitive index, and internalizing problems between autistic and typically developing participants. (a) Represents the simple slopes for the association between social skills and metacognitive functioning among autistic and typically developing participants. Higher scores on social skills and metacognitive functioning indicate poor social skills and higher dysfunction, respectively. (b) Represents the simple slopes for the association between social skills and internalizing problems among autistic and typical participants. Higher scores on social skills and internalizing problems indicate poor social skills and higher levels of internalizing problems, respectively. ASD, autism spectrum disorder, and TD, typical development.

The first model explored whether metacognitive functioning mediates the association between social skills and internalizing problems, and whether this mediating effect varies between groups, using PROCESS Macro Model 59. The overall model was significant (*F* [6, 311] = 69.07; *p* < 0.001) and explained 56.30% of the variance in internalizing problems (see Table 3). The association between social skills and metacognitive functioning significantly varied between autistic and TD participants (a path: *β* [SE] = 0.51 [0.15], *p =* 0.001), such that the effect was stronger in the TD group (*β* [SE] = 1.01 [0.14], *p* < 0.001) compared to the autistic group (*β* [SE] = 0.51 [0.07], *p* < 0.001) (see Figure 2). Metacognition (b path: *β* [SE] = 0.50 [0.22], *p =* 0.023) was found to be associated with internalizing problems. The association between social skills and internalizing problems significantly varied among the diagnostic groups (c′ path: *β* [SE] = 0.41 [0.20], *p =* 0.043), such that the effect was stronger for the TD group (*β* [SE] = 0.70 [0.18], *p =* 0.001) compared to the autistic counterparts (*β* [SE] = 0.29 [0.10], *p =* 0.003) (see Figure 2). Additionally, the results indicated that metacognitive functioning partially mediated the relationship between social skills and internalizing problems among autistic individuals *β* (SE) = 0.17 (0.06), *p* < 0.001 at 95% CI [0.061, 0.280]. However, the indirect effects of metacognitive functioning between social skills and internalizing problems were not significant for the TD participants (see Table 3).

**TABLE 3:** Effects of metacognitive functioning on the relationship between social skills and internalizing problems moderated by diagnosis.

Dependent variable	Independent variable	*β*	SE	*p*	Confidence intervals	
					LLCI	ULCI
Metacognitive functioning						
Social skills		−0.002	0.198	0.992	−0.391	0.289
Diagnosis groups		−0.240	0.138	0.084	−0.512	−0.033
Social skills * Diagnosis		0.508	0.154	0.001	0.206	0.811
FIQ (covariate)		−0.097	0.033	0.004	−0.163	−0.032
Adj. *R* ^2^ = 0.6705, *F* = 162.3, *p* < 0.001						
Internalizing problems						
Social skills		−0.124	0.264	0.637	−0.643	0.394
Metacognitive functioning		0.498	0.217	0.023	0.071	0.926
Diagnosis groups		−0.193	0.163	0.238	−0.514	0.128
Social skills * Diagnosis		0.414	0.203	0.043	0.014	0.814
Metacognitive functioning * Diagnosis		−0.165	0.131	0.211	−0.423	0.094
FIQ (covariate)		0.001	0.039	0.972	−0.076	0.079
Indirect effect: ASD						
Social skills > Metacognitive functioning > Internalizing problems		0.169	0.056	< 0.001	0.061	0.280
Indirect effect: TD						
Social skills > Metacognitive functioning > Internalizing problems		0.172	0.096	> 0.050	−0.025	0.356
Adj. *R* ^2^ = 0.563, *F* = 69.07, *p* < 0.001						

*Note:* Diagnosis groups represent ASD group and TD group.

Abbreviations: ASD, autism spectrum disorder; FIQ, full‐scale intelligence quotient; TD, typical development.

A separate model was used to assess whether behavior regulation mediates the relationship between social skills and externalizing problems, and if this effect is moderated by group differences. The model was statistically significant (*F* [6, 311] = 76.48; *p* < 0.001) and explained 58.82% variance in externalizing problems (see Table 4). It was found that difficulties in social skills were associated with higher behavioral dysregulation (a path: *β* [SE] = 0.43 [0.18], *p =* 0.017). An autism diagnosis was associated with having higher levels of behavioral dysregulation (*β* [SE] = 0.67 [0.07], *p* < 0.001). Behavioral dysregulation was also associated with externalizing problems (b path: *β* [SE] = 0.61 [0.22], *p* = 0.006). Additionally, behavioral dysregulation mediated the relationship between social skills and externalizing problems among the autistic group *β* (SE) = 0.48 (0.09), *p* < 0.001 at 95% CI [0.324, 0.656] and the TD group *β* (SE) = 0.76 (0.15), *p* < 0.001 at 95% CI [0.494, 1.080]. However, this indirect effect did not significantly vary among the autistic and TD groups. See Table 4.

**TABLE 4:** Effects of behavioral regulation on the relationship between social skills and externalizing problems moderated by diagnosis.

Dependent variable	Independent variable	*β*	SE	*p*	Confidence intervals	
					LLCI	ULCI
Behavioral regulation (EF)						
Social skills		0.431	0.179	0.017	0.079	0.783
Diagnosis groups		−0.263	0.125	0.037	−0.509	−0.016
Social skills * Diagnosis		0.237	0.139	0.090	−0.037	0.510
FIQ (covariate)		0.033	0.030	0.270	−0.026	0.093
Adj. *R* ^2^ = 0.7299, *F* = 215.1, *p* < 0.001						
Externalizing problems						
Social skills		−0.309	0.271	0.256	−0.843	0.225
Behavioral regulation		0.607	0.220	0.006	0.175	1.039
Diagnosis group		0.274	0.157	0.082	−0.035	0.582
Social skills * Diagnosis		0.316	0.205	0.123	−0.086	0.718
Behavioral regulation * Diagnosis		0.114	0.140	0.416	−0.162	0.390
FIQ (covariate)		0.015	0.038	0.694	−0.059	0.089
Indirect effect: ASD						
Social skills > Behavioral regulation > Externalizing problems		0.481	0.085	< 0.001	0.324	0.656
Indirect effect: TD						
Social skills > Behavioral regulation > Externalizing problems		0.755	0.150	< 0.001	0.494	1.080
Adj. *R* ^2^ = 0.588, *F* = 76.48, *p* < 0.001						

*Note:* Diagnosis groups represent ASD group, and TD group.

Abbreviations: ASD, autism spectrum disorder; FIQ, full‐scale intelligence quotient; TD, typical development.

Follow up SEM Model 1 demonstrated an acceptable model fit (CFI = 0.932, TLI = 0.912, RMSEA = 0.099, SRMR = 0.126). In the model, latent metacognitive functioning explained 40.2% of variance in the ASD group and 27.0% in the TD group. The measurement model indicated that planning contributed most to metacognitive functioning, followed by working memory, initiation, monitoring and organization among both ASD and TD participants (see Table 5). Additionally, metacognitive functioning had significant indirect effects in the association between social skill deficits and internalizing problems in both groups, with stronger indirect effects in the ASD group (43.56%) compared to the TD group (26.74%). However, the indirect effects did not significantly vary by the diagnostic groups (See Table 6). Specific pathway coefficients for SEM model 1 are presented in Table S1.

**TABLE 5:** Descriptive results of observed variables by diagnostic groups contributing to latent variables in the study.

Observed variables	ASD			TD		
	Mean (SD)	*β*	*p*	Mean (SD)	*β*	*p*
Metacognitive functioning						
Initiation	63.98 (10.24)	0.766	< 0.001	46.11 (8.29)	0.811	< 0.001
Working memory	66.41 (10.46)	0.822	< 0.001	45.47 (7.82)	0.840	< 0.001
Planning	65.56 (11.40)	0.836	< 0.001	45.37 (9.09)	0.891	< 0.001
Organization	59.87 (9.77)	0.603	< 0.001	48.37 (9.66)	0.578	< 0.001
Monitoring	64.85 (10.93)	0.750	< 0.001	44.06 (9.50)	0.774	< 0.001
Behavior regulation index						
Inhibition	62.58 (11.40)	0.569	< 0.001	44.89 (7.01)	0.703	< 0.001
Shifting	68.30 (12.39)	0.692	< 0.001	44.18 (7.59)	0.761	< 0.001
Emotional control	59.80 (11.75)	0.570	< 0.001	44.53 (7.83)	0.669	< 0.001

**TABLE 6:** Result of mediation analysis from structural equation modeling: Metacognitive Functioning as a latent variable.

Outcome: Internalizing problems	ASD	TD	Difference
Predictor: Social skills			
Mediator: Metacognitive functioning			
Indirect effect (a * b)	0.196	0.359	−0.020
SE	0.062	0.044	0.084
*p*‐value	0.002	0.034	0.816
95% CI	(0.095, 0.298)	(0.021, 0.166)	(−0.158, 0.119)
Mediating percentage^a^	43.56%	26.74%	
RID	0.774	0.361	

*Note:* Estimates are standardized. Difference: indicates the moderated mediation effect between the ASD and TD groups.

Abbreviations: ASD, autism spectrum disorder group; CI, confidence interval; SE, standard error; TD, typically developing group.

^a^ Mediating percentage is derived from the ratio of indirect to total effect (RIT); RID is the ratio of indirect to direct effects.

SEM Model 2 demonstrated a poor fit (SEM Model 2 [CFI = 0.897, TLI = −0.820, RMSEA = 0.136, SRMR = 0.139]), indicating misspecification. Therefore, the results must be interpreted with caution. In the model, behavioral dysregulation explained 66.8% of variance in the ASD group and 36.6% in the TD group. The measurement model indicated that shifting contributed most to behavioral dysregulation followed by inhibition and emotional control (See Table 5). Additionally, behavioral dysregulation had significant indirect effects in the association between social skill deficits and internalizing problems in both groups, with stronger indirect effects in the ASD group (262.5%) compared to the TD group (99.52%). However, the indirect effects did not significantly vary by the diagnostic groups (See Table 7). Specific pathway coefficients for SEM Model 2 are presented in Table S2.

**TABLE 7:** Result of mediation analysis from structural equation modeling behavior regulation index as a latent variable.

Outcome: Externalizing problems	ASD	TD	Difference
Predictor: Social skills			
Mediator: Behavior regulation index			
Indirect effect (a * b)	1.029	0.420	0.121
SE	0.308	0.103	0.267
*p*‐value	0.001	0.000	0.650
95% CI	(0.522, 1.537)	(0.250, 0.589)	(−0.318, 0.560)
Mediating percentage^a^	262.5%	99.52%	
RID	−1.615	140	

*Note:* Estimates are standardized. Difference: indicates the moderated mediation effect between the ASD and TD groups.

Abbreviations: ASD, autism spectrum disorder group; CI, Confidence Interval; SE, standard error; TD, typically developing group.

^a^ Mediating percentage is derived from the ratio of indirect to total effect (RIT); RID is the ratio of indirect to direct effects.

## 4 Discussion

The current study examined whether EF, specifically metacognitive functioning and behavioral regulation indices, mediated the relationship between social skill deficits and internalizing and externalizing problems, respectively, and whether these associations varied between autistic and TD children and adolescents. The autistic and TD participants were in their middle childhood and early adolescence, with low‐average to above average cognitive functioning. To our knowledge, this is the first study investigating the pathways underlying the effects of EF and social skills contributing to psychological problems, and whether the pathways differ by diagnostic groups (autistic and TD children and adolescents).

Our findings indicate that, on average, the autistic group had significantly poorer social skills, higher levels of difficulties in metacognitive functioning and poorer behavior regulation skills compared to their TD peers (See Table 1 and Figure S1). Further, as expected, the autistic participants reported higher levels of internalizing and externalizing problems. As hypothesized, poor social skills were found to be associated with higher metacognitive dysregulation, consistent with previous studies (Ameis et al. 2022). However, diverging from our hypothesis, the present results also indicate that this effect is weaker among autistic participants, relative to TD participants. Since these skills were parent‐reported, there is a potential for a systematic bias resulting from lower parental expectations of performance on EF tasks by their autistic compared to TD offspring, which may have attenuated the effect among the autistic participants (Granader et al. 2014). Additionally, this may also be explained by a lesser likelihood among TD children and adolescents (even those with difficulties in social skills or EF) to receive intervention due to personal or structural barriers, as compared to autistic individuals (Radez et al. 2021), which may explain the stronger association between social skill deficits and metacognitive functioning difficulties among TD participants.

The present study also found that metacognitive dysfunction was associated with internalizing problems, highlighting the role of metacognitive functioning in psychological difficulties, which supports our hypothesis and remains consistent with the previous literature (Munsell et al. 2022; Nigg 2017; Nordahl et al. 2023; Warren et al. 2021; Wells and Matthews 2016). As predicted, deficits in social skills were associated with more internalizing problems, supporting previous evidence (Fossum et al. 2023; Tsai et al. 2020). However, contrary to our prediction, this study suggested that these effects were stronger among TD participants. Children and adolescents with poor social skills are more likely to experience social isolation, loneliness, peer‐victimization, and have more psychological problems (Mushtaq et al. 2014; Pavri 2015). Poorer social competence is also associated with diminished lower help‐seeking behaviors, social support, and higher internalizing problems (Pickard et al. 2018; Wole et al. 2025).

Overall, for Model 1, our hypothesis was partially supported, as we found partial mediating effects of metacognition in the association between social skills and internalizing problems only among the autistic participants. Although this finding aligns with previous literature and supports the theoretical metacognitive model of psychopathology (Craig et al. 2016; Munsell et al. 2022; Wang and Liu 2021; Wells and Matthews 2016), it also conflicts with the findings of a previous study (Ameis et al. 2022), where no such significant mediating effect of EF was found among autistic children. It is likely because our study is a retrospective cross‐sectional study, unlike the study by Ameis et al. (2022), which had a longitudinal design. However, it is important to note that Ameis' study (2022) had a smaller sample size and indicated significant missingness in the data, which may have resulted in insignificant effects. Future replication of these associations will help elucidate the role of EF in psychological difficulties in an autistic population. Additionally, contrary to our hypothesis, metacognitive functioning did not explain the relationship between social skills and internalizing problems among the TD participants in our main analyses. Nevertheless, in our exploratory analyses, latent metacognitive functioning, indexed by its subscales on the BRIEF measure, significantly mediated the effects in both the autistic and the TD group. SEM, a highly robust statistical tool, was used for exploratory analyses, which may have accounted for variance that was not captured using the moderated mediation in the main analysis.

For Model 2, which investigated the mediating role of behavior dysregulation in the association between social skill deficits and externalizing problems, and whether this effect was moderated by the diagnostic groups, our findings were partially supported. Autistic group, on average, had significantly higher levels of behavior dysregulation, consistent with previous literature (Dell'Osso et al. 2023). This finding also aligns with the core and associated clinical symptoms of ASD, i.e., social skills deficits and RRB (APA, 2022), which have been explained due to the poorer EF, particularly with respect to skills such as inhibition and difficulty shifting tasks (Iversen and Lewis 2021). As hypothesized, the deficits in social skills were associated with higher levels of behavior dysregulation, supporting previous findings (Fong and Iarocci 2020; Madjar et al. 2019). In addition, behavior dysregulation was associated with externalizing problems, consistent with our predictions and the previous literature (All et al. 2024; Fossum et al. 2023; Perry et al. 2018; Wang and Liu 2021). Finally, our findings support existing evidence for the mediating role of behavioral dysregulation in the association between social skills and externalizing problems among the autistic participants (Ameis et al. 2022) and extend the evidence for this effect among the TD children and adolescents. However, contrary to our prediction, the mediating effect did not significantly vary between the two groups, indicating that, irrespective of the diagnosis, behavior dysregulation plays a role in the association between social skills and externalizing problems, and the pathways do not significantly differ between the two groups. These findings suggest a significant role of self‐regulatory executive processes, such as being able to shift attention and tasks, inhibit responses, and adapting to the environmental demands in increase vulnerabilities to maladaptive behavioral outcomes.

While the overall mediation pathways for both Models 1 and 2 were comparable across ASD and TD groups, as discussed in the preceding paragraphs, the exploratory latent SEM Models 1 and 2 revealed a hierarchically organized EF‐related mechanism underlying the association between social skill deficits and psychological challenges. More specifically, the association between social skill deficits and internalizing challenges was primarily driven by planning and working memory, followed by initiation, monitoring, and organization in both autistic and TD samples. Importantly, autistic participants reported significantly higher loadings for each subscale, suggesting greater difficulties in these EF‐related skills, supporting previous findings (St. John et al. 2026). The findings highlight the role of difficulties in the ability to maintain, manipulate information, and plan goal‐directed behaviors in psychological outcomes.

Exploratory analysis also revealed that the association between deficits in social skills and externalizing behavioral challenges across groups was primarily driven by difficulties in shifting. This finding suggests that cognitive inflexibility is a central component that is commonly associated with behavior regulation. Previous studies highlight the role of cognitive flexibility in behavioral difficulties, including difficulties in RRBs among autistic children (Miller et al. 2015). Interestingly, emotional dysregulation emerged as a secondary contributor in autistic participants, highlighting the role of their emotional challenges. Whereas among TD participants, inhibition emerged as a secondary contributor explaining the indirect effects of behavioral dysregulation, suggesting the role of difficulties inhibiting impulsive responses.

Overall, the findings of this study provide a nuanced understanding of the differential role of the components of EF (metacognitive functioning and behavioral regulation) in internalizing and externalizing problems in autistic and TD individuals. Our findings indicate that metacognitive functioning mediated the association between social skills and internalizing problems only among the autistic participants, whereas behavioral dysregulation mediated a similar relationship in both autistic and the TD participants. While we predicted the mediating effects in both models to significantly vary between the diagnostic groups, we found that the mediating effects of EF were not statistically different. This is a notable finding as it supports the emerging transdiagnostic framework emphasizing the role of EF as a dimensional process rather than a set of skills specifically associated as characterized in specific diagnostic groups, ASD in this case.

The findings of this study are promising, though a few limitations should be acknowledged. This is a retrospective cross‐sectional study, in which data from four different sites were used, which may potentially introduce several forms of between‐sites variability, including differences in assessment procedures. Future studies may carry out a prospective, longitudinal study and explore a different pool of data with less variability. Another key limitation is that due to the lack of sufficient data, we could not control for the severity of autistic symptoms and potential interventions the autistic participants were receiving. These are important factors that are associated with levels of functioning, psychological outcomes and reported EF skills. Further, the study did not control for co‐occurring conditions among the ASD group, due to lack of sufficient data. EF deficits are highly prevalent among individuals with several disorders, such as attention deficit‐hyperactivity disorder (ADHD) (Roselló et al. 2020), which highly co‐occurs with ASD. Future studies should address this and control for co‐occurring diagnoses and isolate the effects of executive dysfunction. Additionally, since our participants are children and adolescents, the measures used were parent/guardian reported and not observational. This can potentially introduce response bias, though this was addressed through assessment of multicollinearity as described in the Methods section. However, the BRIEF is a caregiver reported measure of EF skills, as opposed to an objective task‐based measure of pure cognitive capacity EF. Additionally, BRIEF has been found to be uncorrelated or poorly correlated with performance‐based tests of EF, including the Wisconsin Card Sorting Test, Trail Making Test, Stop‐Signal task of inhibition and monitoring, and several other performance‐based EF tasks (McAuley et al. 2010; Snyder et al. 2021; Vriezen and Pigott 2002). Studies have also reported that BRIEF is sensitive to behavioral difficulties and performance (McAuley et al. 2010). Therefore, the interpretation of results must consider the EF‐related construct measured in this study. Future studies may include performance‐based EF task measures to assess purely cognitive EF abilities. Studies may specifically focus on the different skills of EF to precisely identify the core challenge associated with specific types of psychological challenges. The relationship between social skills and metacognitive dysfunction, internalizing and externalizing problems among TD children and adolescents must be investigated further to clarify the stronger associations among the TD participants compared to their autistic counterparts.

Understanding the pathways for internalizing and externalizing problems is important for identifying at‐risk youth and for developing more accurate assessments and effective interventions for these problems. The current findings emphasize the important role of EF and its dysfunction in psychological problems during middle childhood and early adolescence. This is particularly true for autistic individuals, where both metacognitive functioning and behavioral dysregulation mediated the relationship between social skills and internalizing and externalizing problems, respectively. On the other hand, only behavioral dysregulation mediated the effects of social skills and externalizing problems among the TD participants. The two groups were not different in how EF played a role in this association. These findings may help early identification of children and adolescents who are vulnerable to developing internalizing and externalizing problems, based on their social skills and executive functioning. The current findings may also inform interventions for autistic individuals experiencing internalizing and externalizing problems, where metacognitive skills and behavioral regulation skills may be taught to children and adolescents with ASD. For TD children and adolescents, interventions for externalizing problems may focus on developing behavioral regulation skills. Future studies should continue to investigate the differential role of EF in psychopathology among autistic, TD, as well as other diagnostic groups, to investigate the dimensional effects of EF in psychological challenges, independent of diagnosis.

## Author Contributions

**Samina I. Ansari:** conceptualization, literature search, methodology, data extraction, analysis, writing original draft. **Lucas A. Martin:** conceptualization, literature search, data analysis, reviewing and editing. **Kristi C. Guest:** supervision, methodology, reviewing. **Rajesh K. Kana:** conceptualization, methodology, supervision, reviewing, and editing.

## Funding

This research was supported by the University of Alabama at Birmingham (UAB) College of Arts and Sciences Faculty Funds.

## Ethics Statement

All methods were carried out in accordance with the relevant guidelines and regulations of the UAB Institutional Review Board. The participants at each site for the ABIDE‐II data provided their informed consent to participate in this study.

## Consent

All authors provided consent for publication.

## Conflicts of Interest

The authors declare no conflicts of interest.

## Supporting information


**Figure S1:** Mean differences among autistic and typically developing participants The figure represents mean differences in the predictor and outcome variables across the diagnostic groups. ASD, autism spectrum disorder, and TD, typical development; FIQ, full‐scale intelligence quotient.

**Table S1:** Path coefficient estimates and significance level from SEM model, metacognitive functioning as a latent variable.
**Table S2:** Path coefficient estimates and significance level from SEM model, behavior regulation index as a latent variable.

## Data Availability

The study used a dataset that is available online in the public domain. For more details, please visit: https://fcon_1000.projects.nitrc.org/indi/abide/abide_II.html.

## References

[aur70305-bib-0001] Achenbach, T. M. 1966. “The Classification of Children's Psychiatric Symptoms: A Factor‐Analytic Study.” Psychological Monographs: General and Applied 80, no. 7: 1–37. 10.1037/h0093906 5968338

[aur70305-bib-0002] Achenbach, T. M. , L. Dumenci , and L. A. Rescorla . 2003. “Are American Children's Problems Still Getting Worse? A 23‐Year Comparison.” Journal of Abnormal Child Psychology 31: 1–11. 12597695 10.1023/a:1021700430364

[aur70305-bib-0003] Achenbach, T. M. , and L. A. Rescorla . 2001. Manual for the ASEBA School‐Age Forms & Profiles. University of Vermont, Research Center for Children, Youth, & Families.

[aur70305-bib-0004] All, K. , K. Chawarska , and S. L. Macari . 2024. “Early Executive Functioning Predicts Externalizing Problems in Neurodiverse Preschoolers.” Autism Research 17, no. 5: 1053–1065. 10.1002/aur.3109. 38476104 PMC11251695

[aur70305-bib-0005] Ameis, S. H. , J. D. Haltigan , R. E. Lyon , et al. 2022. “Middle‐Childhood Executive Functioning Mediates Associations Between Early‐Childhood Autism Symptoms and Adolescent Mental Health, Academic and Functional Outcomes in Autistic Children.” Journal of Child Psychology and Psychiatry, and Allied Disciplines 63, no. 5: 553–562. 10.1111/jcpp.13493. 34382216 PMC9291328

[aur70305-bib-0006] American Psychiatric Association . 2022. Diagnostic and Statistical Manual of Mental Disorders. 5th ed. American Psychiatric Association. 10.1176/appi.books.9780890425787.

[aur70305-bib-0007] Arslan, İ. B. , N. Lucassen , P. A. C. Van Lier , A. D. De Haan , and P. Prinzie . 2021. “Early Childhood Internalizing Problems, Externalizing Problems and Their Co‐Occurrence and (Mal)adaptive Functioning in Emerging Adulthood: A 16‐Year Follow‐Up Study.” Social Psychiatry and Psychiatric Epidemiology 56, no. 2: 193–206. 10.1007/s00127-020-01959-w. 32964254 PMC7870752

[aur70305-bib-0008] Bijstra, J. O. , and S. Jackson . 1998. “Social Skills Training With Early Adolescents: Effects on Social Skills, Well‐Being, Self‐Esteem and Coping.” European Journal of Psychology of Education 13, no. 4: 569–583. 10.1007/bf03173106.

[aur70305-bib-0009] Blakemore, S.‐J. , and K. L. Mills . 2014. “Is Adolescence a Sensitive Period for Sociocultural Processing?” Annual Review of Psychology 65, no. 1: 187–207. 10.1146/annurev-psych-010213-115202. 24016274

[aur70305-bib-0010] Boettcher, J. , S. Orm , and K. W. Fjermestad . 2024. “Autism Traits, Social Withdrawal, and Behavioral and Emotional Problems in a Norwegian Cohort of Adolescents With Rare Genetic Disorders.” Research in Developmental Disabilities 147: 104699. 10.1016/j.ridd.2024.104699. 38367299

[aur70305-bib-0011] Bornstein, M. H. , C. S. Hahn , and O. M. Haynes . 2010. “Social Competence, Externalizing, and Internalizing Behavioral Adjustment From Early Childhood Through Early Adolescence: Developmental Cascades.” Development and Psychopathology 22, no. 4: 717–735. 10.1017/s0954579410000416. 20883577 PMC3412561

[aur70305-bib-0012] Constantino, J. N. , and C. P. Gruber . 2005. The Social Responsiveness Scale. Western Psychological Services.

[aur70305-bib-0013] Constantino, J. N. , and C. P. Gruber . 2012. The Social Responsiveness Scale. 2nd ed. Western Psychological Services.

[aur70305-bib-0032] Craig, F. , F. Margari , A. R. Legrottaglie , R. Palumbi , C. de Giambattista , and L. Margari . 2016. “A Review of Executive Function Deficits in Autism Spectrum Disorder and Attention‐Deficit/Hyperactivity Disorder.” Neuropsychiatric Disease and Treatment 12: 1191–1202. 10.2147/ndt.s104620. 27274255 PMC4869784

[aur70305-bib-0014] Dell'Osso, L. , L. Massoni , S. Battaglini , et al. 2023. “Emotional Dysregulation as a Part of the Autism Spectrum Continuum: A Literature Review From Late Childhood to Adulthood.” Frontiers in Psychiatry 14: 1234518. 10.3389/fpsyt.2023.1234518. 37791135 PMC10544895

[aur70305-bib-0015] Demetriou, E. A. , A. Lampit , D. S. Quintana , et al. 2018. “Autism Spectrum Disorders: A Meta‐Analysis of Executive Function.” Molecular Psychiatry 23, no. 5: 1198–1204. 10.1038/mp.2017.75. 28439105 PMC5984099

[aur70305-bib-0016] Donoso, J. , F. Rattray , A. De Bildt , et al. 2024. “Association of Cognitive and Adaptive Skills With Internalizing and Externalizing Problems in Autistic Children and Adolescents.” Autism Research 17, no. 3: 596–609. 10.1002/aur.3056. 38031634

[aur70305-bib-0017] Fong, V. C. , and G. Iarocci . 2020. “The Role of Executive Functioning in Predicting Social Competence in Children With and Without Autism Spectrum Disorder.” Autism Research 13, no. 11: 1856–1866. 10.1002/aur.2350. 33460309

[aur70305-bib-0018] Fossum, I. N. , S. Orm , P. N. Andersen , H. M. Geurts , M. G. Øie , and E. W. Skogli . 2023. “Childhood Executive Function Predicts Internalizing and Externalizing Symptoms in Emerging Adults With and Without Autism: A 10‐Year Longitudinal Study.” Developmental Neuropsychology 48, no. 3: 97–111. 10.1080/87565641.2023.2206663. 37154789

[aur70305-bib-0019] Freichel, R. , J. Pfirrmann , P. J. De Jong , et al. 2024. “Executive Functioning, Internalizing and Externalizing Symptoms: Understanding Developmental Dynamics Through Panel Network Approaches.” JAACAP Open 2, no. 1: 66–77. 10.1016/j.jaacop.2023.11.001. 39554700 PMC11562421

[aur70305-bib-0020] Frogner, L. , K. Hellfeldt , A.‐K. Ångström , et al. 2022. “Stability and Change in Early Social Skills Development in Relation to Early School Performance: A Longitudinal Study of A Swedish Cohort.” Early Education and Development 33, no. 1: 17–37. 10.1080/10409289.2020.1857989.

[aur70305-bib-0021] Fuhrmann, D. , L. J. Knoll , and S.‐J. Blakemore . 2015. “Adolescence as a Sensitive Period of Brain Development.” Trends in Cognitive Sciences 19, no. 10: 558–566. 26419496 10.1016/j.tics.2015.07.008

[aur70305-bib-0022] Gilotty, L. , L. Kenworthy , L. Sirian , D. O. Black , and A. E. Wagner . 2002. “Adaptive Skills and Executive Function in Autism Spectrum Disorders.” Child Neuropsychology 8, no. 4: 241–248. 12759821 10.1076/chin.8.4.241.13504

[aur70305-bib-0023] Gioia, G. , P. Isquith , S. C. Guy , and L. Kenworthy . 2000. “Behavior Rating Inventory of Executive Function.” Child Neuropsychology 6: 235–238. 11419452 10.1076/chin.6.3.235.3152

[aur70305-bib-0024] González, L. L. , and L. M. L. Fernández . 2017. “Los trastornos internalizantes: un reto para padres y docentes.” Padres y Maestros/Journal of Parents and Teachers 372: 56–63.

[aur70305-bib-0025] Granader, Y. , G. L. Wallace , K. K. Hardy , et al. 2014. “Characterizing the Factor Structure of Parent Reported Executive Function in Autism Spectrum Disorders: The Impact of Cognitive Inflexibility.” Journal of Autism and Developmental Disorders 44, no. 12: 3056–3062. 10.1007/s10803-014-2169-8. 24972681 PMC6084425

[aur70305-bib-0026] Hayes, A. F. 2017. Introduction to Mediation, Moderation, and Conditional Process Analysis, Second Edition: A Regression‐Based Approach. Guilford Publications.

[aur70305-bib-0027] Hill, E. L. 2004. “Evaluating the Theory of Executive Dysfunction in Autism.” Developmental Review 24, no. 2: 189–233. 10.1016/j.dr.2004.01.001.

[aur70305-bib-0028] Hughes, C. , J. Russell , and T. W. Robbins . 1994. “Evidence for Executive Dysfunction in Autism.” Neuropsychologia 32, no. 4: 477–492. 10.1016/0028-3932(94)90092-2. 8047253

[aur70305-bib-0029] Hunduma, G. , Y. Dessie , B. Geda , T. A. Yadeta , and N. Deyessa . 2024. “Prevalence and Correlates of Internalizing and Externalizing Mental Health Problems Among in‐School Adolescents in Eastern Ethiopia: A Cross‐Sectional Study.” Scientific Reports 14, no. 1: 3574. 10.1038/s41598-024-54145-2. 38347112 PMC10861546

[aur70305-bib-0030] Iversen, R. K. , and C. Lewis . 2021. “Executive Function Skills Are Linked to Restricted and Repetitive Behaviors: Three Correlational Meta Analyses.” Autism Research 14, no. 6: 1163–1185. 10.1002/aur.2468. 33410263

[aur70305-bib-0031] Madjar, N. , E. Chubarov , G. Zalsman , M. Weiser , and G. Shoval . 2019. “Social Skills, Executive Functioning and Social Engagement.” Schizophr Res Cogn 17: 100137. 10.1016/j.scog.2019.100137. 31024800 PMC6476806

[aur70305-bib-0033] McAuley, T. , S. Chen , L. Goos , R. Schachar , and J. Crosbie . 2010. “Is the Behavior Rating Inventory of Executive Function More Strongly Associated With Measures of Impairment or Executive Function?” Journal of the International Neuropsychological Society 16, no. 3: 495–505. 10.1017/s1355617710000093. 20188014

[aur70305-bib-0034] Mezzacappa, E. 2011. “Executive Function.” In Encyclopedia of Adolescence, edited by B. B. Brown and M. J. Prinstein , 142–150. Academic Press. 10.1016/B978-0-12-373951-3.00016-8.

[aur70305-bib-0035] Miller, H. L. , M. E. Ragozzino , E. H. Cook , J. A. Sweeney , and M. W. Mosconi . 2015. “Cognitive Set Shifting Deficits and Their Relationship to Repetitive Behaviors in Autism Spectrum Disorder.” Journal of Autism and Developmental Disorders 45, no. 3: 805–815. 10.1007/s10803-014-2244-1. 25234483 PMC4658328

[aur70305-bib-0036] Munsell, E. G. S. , G. I. Orsmond , D. Fulford , and W. J. Coster . 2022. “Metacognition Mediates the Effect of Social Communication and Internalizing Behaviors on Self‐Management of Daily Life Tasks for Diploma‐Track Autistic Youth.” Journal of Autism and Developmental Disorders 52, no. 10: 4274–4285. 10.1007/s10803-021-05306-z. 34611837

[aur70305-bib-0037] Mushtaq, R. , S. Shoib , T. Shah , and S. Mushtaq . 2014. “Relationship Between Loneliness, Psychiatric Disorders and Physical Health ? A Review on the Psychological Aspects of Loneliness.” Journal of Clinical and Diagnostic Research 8, no. 9: We01–We04. 10.7860/jcdr/2014/10077.4828. 25386507 PMC4225959

[aur70305-bib-0038] Nigg, J. T. 2017. “Annual Research Review: On the Relations Among Self‐Regulation, Self‐Control, Executive Functioning, Effortful Control, Cognitive Control, Impulsivity, Risk‐Taking, and Inhibition for Developmental Psychopathology.” Journal of Child Psychology and Psychiatry 58, no. 4: 361–383. 10.1111/jcpp.12675. 28035675 PMC5367959

[aur70305-bib-0039] Nikstat, A. , and R. Riemann . 2020. “On the Etiology of Internalizing and Externalizing Problem Behavior: A Twin‐Family Study.” PLoS One 15, no. 3: e0230626. 10.1371/journal.pone.0230626. 32203544 PMC7089526

[aur70305-bib-0040] Nordahl, H. , F. Anyan , and O. Hjemdal . 2023. “Prospective Relations Between Dysfunctional Metacognitive Beliefs, Metacognitive Strategies, and Anxiety: Results From a Four‐Wave Longitudinal Mediation Model.” Behavior Therapy 54, no. 5: 765–776. 10.1016/j.beth.2023.02.003. 37597956

[aur70305-bib-0041] Ogden, T. 2015. Sosial kompetanse og problematferd blant barn og unge. Gyldendal akademisk.

[aur70305-bib-0042] Pavri, S. 2015. “Loneliness: The Cause or Consequence of Peer Victimization in Children and Youth.” Open Psychology Journal 8, no. 1: 78–84. 10.2174/1874350101508010078.

[aur70305-bib-0043] Pennington, B. F. , and S. Ozonoff . 1996. “Executive Functions and Developmental Psychopathology.” Journal of Child Psychology and Psychiatry 37, no. 1: 51–87. 10.1111/j.1469-7610.1996.tb01380.x. 8655658

[aur70305-bib-0044] Perry, N. B. , S. D. Calkins , J. M. Dollar , S. P. Keane , and L. Shanahan . 2018. “Self‐Regulation as a Predictor of Patterns of Change in Externalizing Behaviors From Infancy to Adolescence.” Development and Psychopathology 30, no. 2: 497–510. 10.1017/s0954579417000992. 28641597 PMC5858969

[aur70305-bib-0045] Pickard, H. , F. Happé , and W. Mandy . 2018. “Navigating the Social World: The Role of Social Competence, Peer Victimisation and Friendship Quality in the Development of Social Anxiety in Childhood.” Journal of Anxiety Disorders 60: 1–10. 10.1016/j.janxdis.2018.09.002. 30268999 PMC6269163

[aur70305-bib-0046] Radez, J. , T. Reardon , C. Creswell , P. J. Lawrence , G. Evdoka‐Burton , and P. Waite . 2021. “Why Do Children and Adolescents Seek and Access Professional Help for Their Mental Health Problems? A Systematic Review of Quantitative and Qualitative Studies.” European Child & Adolescent Psychiatry 30, no. 2: 183–211. 10.1007/s00787-019-01469-4. 31965309 PMC7932953

[aur70305-bib-0047] Razza, R. A. , and C. Blair . 2009. “Associations Among False‐Belief Understanding, Executive Function, and Social Competence: A Longitudinal Analysis.” Journal of Applied Developmental Psychology 30, no. 3: 332–343. 20161159 10.1016/j.appdev.2008.12.020 PMC2735755

[aur70305-bib-0048] Roselló, B. , C. Berenguer , I. Baixauli , Á. Mira , J. Martinez‐Raga , and A. Miranda . 2020. “Empirical Examination of Executive Functioning, ADHD Associated Behaviors, and Functional Impairments in Adults With Persistent ADHD, Remittent ADHD, and Without ADHD.” BMC Psychiatry 20, no. 1: 134. 10.1186/s12888-020-02542-y. 32204708 PMC7092442

[aur70305-bib-0049] Sari, N. P. , M. P. C. M. Luijk , P. Prinzie , M. H. Van Ijzendoorn , and P. W. Jansen . 2021. “Children's Autistic Traits and Peer Relationships: Do Non‐Verbal IQ and Externalizing Problems Play a Role?” Child and Adolescent Psychiatry and Mental Health 15, no. 1: 67. 10.1186/s13034-021-00421-2. 34809682 PMC8609782

[aur70305-bib-0050] Segrin, C. , A. Hanzal , C. Donnerstein , M. Taylor , and T. Domschke . 2007. “Social Skills, Psychological Well‐Being, and the Mediating Role of Perceived Stress.” Anxiety, Stress, and Coping 20, no. 3: 321–329. 10.1080/10615800701282252. 17999232

[aur70305-bib-0051] Selemon, L. D. 2013. “A Role for Synaptic Plasticity in the Adolescent Development of Executive Function.” Translational Psychiatry 3, no. 3: e238. 10.1038/tp.2013.7. 23462989 PMC3625918

[aur70305-bib-0052] Snyder, H. R. , N. P. Friedman , and B. L. Hankin . 2021. “Associations Between Task Performance and Self‐Report Measures of Cognitive Control: Shared Versus Distinct Abilities.” Assessment 28, no. 4: 1080–1096. 10.1177/1073191120965694. 33084353 PMC8058111

[aur70305-bib-0053] Sørlie, M.‐A. , K. A. Hagen , and K. B. Nordahl . 2021. “Development of Social Skills During Middle Childhood: Growth Trajectories and School‐Related Predictors.” International Journal of School & Educational Psychology 9, no. sup1: S69–S87. 10.1080/21683603.2020.1744492.

[aur70305-bib-0054] St. John, T. , N. M. Corrigan , A. Rokem , et al. 2026. “Longitudinal Profiles of Executive Function in Autistic and Non‐Autistic Children at High Likelihood of Autism.” Journal of Neurodevelopmental Disorders 18, no. 1: 21. 10.1186/s11689-026-09682-4. 41820855 PMC13093929

[aur70305-bib-0055] Tsai, C.‐H. , K.‐L. Chen , H.‐J. Li , et al. 2020. “The Symptoms of Autism Including Social Communication Deficits and Repetitive and Restricted Behaviors Are Associated With Different Emotional and Behavioral Problems.” Scientific Reports 10, no. 1: 20509. 10.1038/s41598-020-76292-y. 33239663 PMC7689452

[aur70305-bib-0056] Vriezen, E. R. , and S. E. Pigott . 2002. “The Relationship Between Parental Report on the BRIEF and Performance‐Based Measures of Executive Function in Children With Moderate to Severe Traumatic Brain Injury.” Child Neuropsychology 8, no. 4: 296–303. 10.1076/chin.8.4.296.13505. 12759826

[aur70305-bib-0057] Wang, Y. , and Y. Liu . 2021. “The Development of Internalizing and Externalizing Problems in Primary School: Contributions of Executive Function and Social Competence.” Child Development 92, no. 3: 889–903. 10.1111/cdev.13462. 32857446

[aur70305-bib-0058] Warren, S. L. , W. Heller , and G. A. Miller . 2021. “The Structure of Executive Dysfunction in Depression and Anxiety.” Journal of Affective Disorders 279: 208–216. 10.1016/j.jad.2020.09.132. 33059224 PMC7738359

[aur70305-bib-0059] Wells, A. , and G. Matthews . 2016. Attention and Emotion: A Clinical Perspective. Psychology Press.

[aur70305-bib-0060] Wole, F. T. , R. D. Negasi , and A. S. Abebe . 2025. “Academic Help Seeking Behavior as a Mediator of the Relationship Between Social Skill and Mathematics Achievement Among Primary School Students.” Scientific Reports 15, no. 1: 19533. 10.1038/s41598-025-99014-8. 40467933 PMC12137582

[aur70305-bib-0061] Yang, Y. , G. S. Shields , Y. Zhang , H. Wu , H. Chen , and A. L. Romer . 2022. “Child Executive Function and Future Externalizing and Internalizing Problems: A Meta‐Analysis of Prospective Longitudinal Studies.” Clinical Psychology Review 97: 102194. 10.1016/j.cpr.2022.102194. 35964337

[aur70305-bib-0062] Zelazo, P. D. 2020. “Executive Function and Psychopathology: A Neurodevelopmental Perspective.” Annual Review of Clinical Psychology 16, no. 1: 431–454. 10.1146/annurev-clinpsy-072319-024242. 32075434

