# Supplementary material for: Executive Functioning as a Pathway Between Social Skills and Psychopathology: The Moderating Role of Autism Diagnosis
Source: Autism Res. 2026 Jun 26;19(8):e70305. doi: 10.1002/aur.70305 (PMC13471877; doi:10.1002/aur.70305)
Supplement: Supplementary file 1 [file AUR-19-0-s001.pdf]

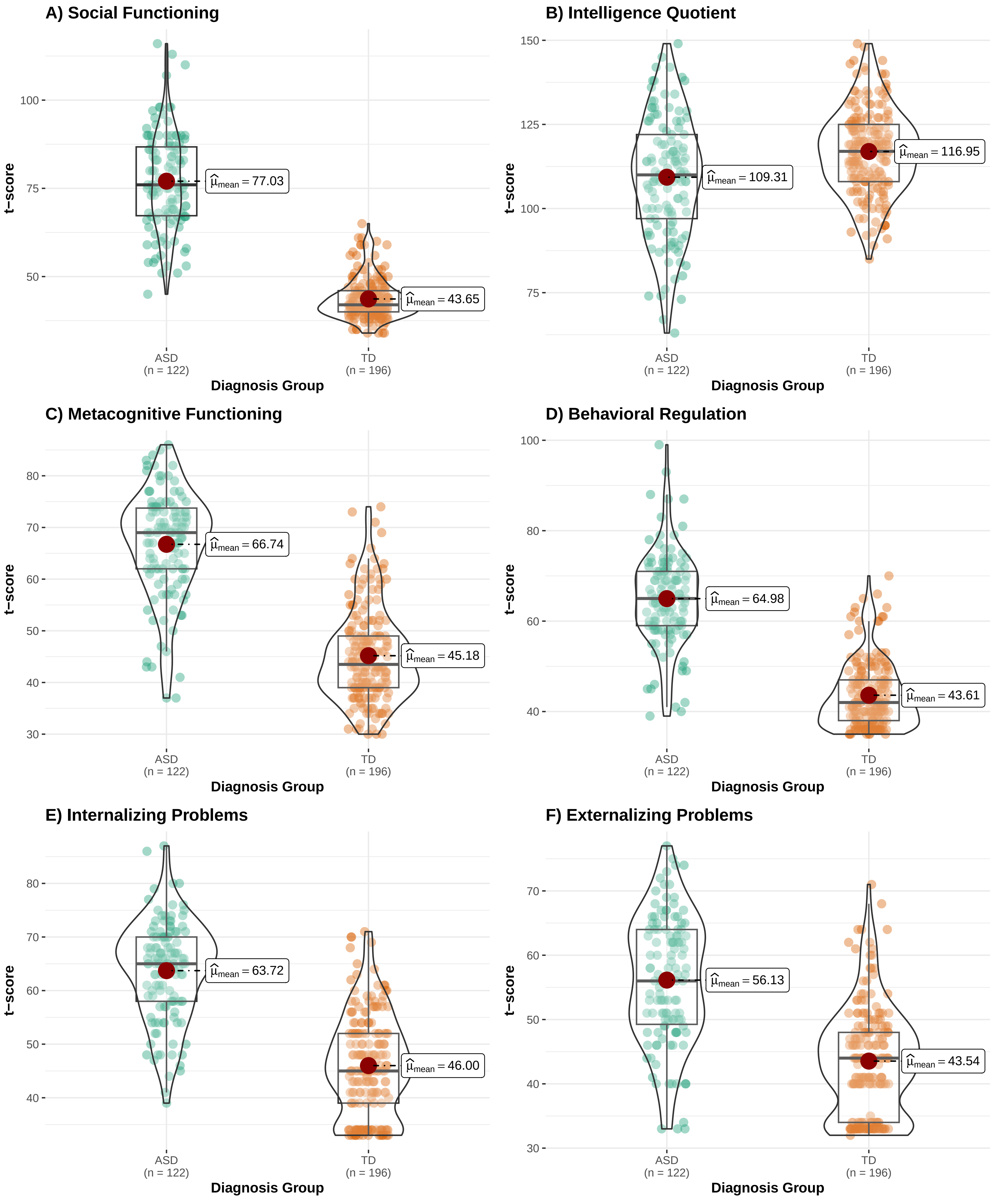
A) Social Functioning
B) Intelligence Quotient
C) Metacognitive Functioning
D) Behavioral Regulation
E) Internalizing Problems
F) Externalizing Problems
t−score
$\widehat{\mu}_{mean} = 77.03$
$\widehat{\mu}_{mean} = 43.65$
$\widehat{\mu}_{mean} = 109.31$
$\widehat{\mu}_{mean} = 116.95$
$\widehat{\mu}_{mean} = 66.74$
$\widehat{\mu}_{mean} = 45.18$
$\widehat{\mu}_{mean} = 64.98$
$\widehat{\mu}_{mean} = 43.61$
$\widehat{\mu}_{mean} = 63.72$
$\widehat{\mu}_{mean} = 46.00$
$\widehat{\mu}_{mean} = 56.13$
$\widehat{\mu}_{mean} = 43.54$
ASD
(n = 122)
TD
(n = 196)
Diagnosis Group
