# Supplementary material for: Executive Functioning as a Pathway Between Social Skills and Psychopathology: The Moderating Role of Autism Diagnosis
Source: Autism Res. 2026 Jun 26;19(8):e70305. doi: 10.1002/aur.70305 (PMC13471877; doi:10.1002/aur.70305)
Supplement: Supplementary file 2 [file AUR-19-0-s002.docx]

**Table S1:** Path coefficient estimates and significance level from SEM model, metacognitive functioning as a latent variable.

| **Path** | **ASD** | | | **TD** | | |
| --- | --- | --- | --- | --- | --- | --- |
|  | **β** | **SE** | **CR** | **β** | **SE** | **CR** |
| Social Skills ® Metacognitive Functioning (a) | 0.541*** | 0.066 | 8.20 | 0.520*** | 0.071 | 7.32 |
| Metacognitive Functioning ® Internalizing Problems (b) | 0.363*** | 0.103 | 3.52 | 0.180* | 0.082 | 2.20 |
| Social Skills ® Internalizing Problems (c’) | 0.253** | 0.092 | 2.75 | 0.266*** | 0.075 | 3.55 |
| FIQ ® Metacognitive Functioning | -0.294*** | 0.083 | -3.54 | -0.018 | 0.059 | -0.31 |
| FIQ ® Internalizing Problems | -0.028 | 0.087 | -0.32 | 0.047 | 0.069 | 0.68 |
| R2- Metacognitive Functioning (latent) | 0.402*** | 0.071 | 5.66 | 0.270*** | 0.074 | 3.65 |

*Estimate is standardized coefficients; SE is Standard Error; CR is Critical Ratio and calculated by dividing the estimate by its standard error; *p<0.001. CFI = 0.932, TLI = 0.912, SRMR = 0.126*

**Table S2:** Path coefficient estimates and significance level from SEM model, behavior regulation index as a latent variable.

| **Path** | **ASD** | | | **TD** | | |
| --- | --- | --- | --- | --- | --- | --- |
|  | **β** | **SE** | **CR** | **β** | **SE** | **CR** |
| Social Skills ® Behavior Regulation Index (a) | 0.811*** | 0.057 | 14.23 | 0.580*** | 0.073 | 7.95 |
| Behavior Regulation Index ® Externalizing Problems (b) | 1.269*** | 0.308 | 4.12 | 0.724*** | 0.110 | 6.58 |
| Social Skills ® Externalizing Problems (c’) | -0.637** | 0.310 | -2.05 | 0.003 | 0.111 | 0.03 |
| FIQ ® Behavior Regulation Index | -0.056 | 0.079 | -0.71 | 0.163 | 0.061 | 2.67 |
| FIQ ® Externalizing Problems | 0.121 | 0.084 | 1.44 | -0.065 | 0.066 | -0.98 |
| R2- Behavior Regulation Index (latent) | 0.668*** | 0.092 | 7.26 | 0.366*** | 0.084 | 4.36 |

*Estimate is standardized coefficients; SE is Standard Error; CR is Critical Ratio and calculated by dividing the estimate by its standard error; *p<0.001. CFI = 0.897, TLI = -0.820, SRMR = 0.139*
